# Preparation and characterization of RhFe/g-C_3_N_4_ nanoparticles for efficient hydrolysis of sodium borohydride

**DOI:** 10.55730/1300-0527.3711

**Published:** 2024-12-11

**Authors:** Merve UÇAR KAYA, Mehmet YURDERİ, Mehmet ZAHMAKIRAN

**Affiliations:** 1 Department of Metallurgical and Materials Engineering, Faculty of Engineering, Architecture and Design, University of Bartın, Bartın, Turkiye; 2 Department of Electronics and Automation, Bartın Vocational School, University of Bartın, Bartın, Turkiye; 3 Central Research Laboratory, Research & Application Center, NanoMatCat Research Laboratory, University of Bartın, Bartın, Turkiye; 4 Department of Biotechnology, Faculty of Science, Bartın University, Bartın, Turkiye

**Keywords:** Sodium borohydride, hydrolysis, graphitic carbon nitride, catalyst, rhodium, iron

## Abstract

Highly effective graphitic carbon nitride-supported RhFe nanoparticles (RhFe/g-C_3_N_4_) were prepared using a simple wet impregnation-reduction method for the hydrolysis of sodium borohydride (NaBH_4_). The obtained Rh_0.48_Fe_0.52_/g-C_3_N_4_ catalyst was characterized using advanced analytical/spectroscopic techniques such as inductively coupled plasma-optical emission spectroscopy, scanning electron microscopy, transmission electron microscopy, powder X-ray diffraction, X-ray spectroscopy, and Fourier transform infrared spectroscopy. The catalytic activities of RhFe/g-C_3_N_4_ catalysts with varying Rh/Fe molar ratios were investigated, demonstrating superior catalytic activity compared to monometallic Rh and Fe. The initial turnover frequency value of the Rh_0.48_Fe_0.52_/g-C_3_N_4_ catalyst was calculated as 33.04 min^−1^ and it showed an H_2_ generation rate of $4214.02\;\text{mL }{\text{min}}^{-1}\;{\text{g}}_{\text{cat}}^{-1}$ at 298 K. Furthermore, the kinetic parameters (*E*_a_^#^, Δ*H*^#^, Δ*S*^#^) of the hydrolysis of NaBH_4_ catalyzed by the Rh_0.48_Fe_0.52_/g-C_3_N_4_ catalyst were elucidated. The findings demonstrated that the resulting Rh_0.48_Fe_0.52_/g-C_3_N_4_ catalyst was recyclable and retained 80.72% of the initial activity even after 10 runs.

## 1. Introduction

One of the scenarios for reducing carbon dioxide (CO_2_) emissions, which contribute to climate change due to fossil fuel consumption, involves using renewable energy and transitioning to a hydrogen economy without carbon emissions. The challenges of this transition include storage and security concerns [1,2]. Among these, boron compounds are promising solid hydrogen (H_2_) storage materials that are widely studied in academia for H_2_ production [3,4]. Sodium borohydride (NaBH_4_) has garnered considerable interest from researchers as a solid hydrogen storage material due to its notable attributes, including a sizable theoretical gravimetric H_2_ storage capacity of 10.8 wt% (or volumetric H_2_ storage capacity of 115 kg m^−3^), cost-effectiveness, nontoxicity, stability, nonflammability, favorable reaction conditions, ease of storage, and environmentally friendly byproducts upon release [5,6]. Conventional methods for releasing the H_2_ stored in NaBH_4_ include pyrolysis and hydrolysis. Generally, pyrolysis requires a high temperature (~400°C), leading to high energy consumption unsuitable for practical applications. However, with suitable catalysts, the hydrolysis of NaBH_4_ easily releases 4 moles of H_2_, even at room temperature Eq. (1) [5,7,8]:


(1) $${\text{NaBH}}_{4}+2{\text{H}}_{2}\text{O}\to {\text{NaBO}}_{2}(\text{aq})+4{\text{H}}_{2}$$

A low-cost catalyst with high catalytic efficiency and stability is vital in practical fuel cell applications. Recent research has focused on developing bimetallic catalysts incorporating cheaper, abundant first-row transition metals to reduce the consumption of expensive precious metals. Employing nonprecious metals to prepare bimetallic catalysts reduces the cost and secondary precious metal content [9–11]. These bimetallic catalysts often exhibit enhanced catalytic efficiency compared to monometallic catalysts due to synergistic effects [12,13].

The nanoparticles (NPs) in the catalyst tend to aggregate into larger particles, blocking active sites and reducing catalytic performance, especially during recyclability tests. Therefore, solid support materials are essential to prevent active site blockage and metal leaching. In heterogeneous catalysis, the interaction between metal NPs and support materials positively enhances the catalyst’s performance [14–17]. Graphitic carbon nitride (g-C_3_N_4_), a two-dimensional (2D), metal-free organic polymer material, can be easily prepared via pyrolysis reaction and emerges as an interesting solid support material for stabilizing NPs. To the best of our knowledge, g-C_3_N_4_ is used in many application areas, such as H_2_ production [18], pollutant degradation [19], water splitting [20], and hydrogen peroxide production [21], owing to its affordability, environmental friendliness, and unique physicochemical and structural properties [22,23]. Therefore, substantial effort is required to investigate the utilization of g-C_3_N_4_ as a solid support material.

The motivation herein was to develop stable and efficient bimetallic catalysts for NaBH_4_ hydrolysis, combining precious metals such as Pt, Ru, and Rh with nonprecious metals like Ni, Co, Cu, and Fe. Hence, this study presents g-C_3_N_4_-supported rhodium/iron (RhFe) (RhFe/g-C_3_N_4_) NPs via the impregnation-reduction method and H_2_ production in the hydrolysis of NaBH_4_ at 298 K. Moreover, the obtained catalyst was characterized by advanced analytical methods like inductively coupled plasma-optical emission spectroscopy (ICP-OES), scanning electron microscopy (SEM), electron microscopy (TEM), powder X-ray diffraction (P-XRD), X-ray spectroscopy (XPS), and Fourier transform infrared spectroscopy (FTIR).

## 2. Materials and methods

### 2.1. Chemicals

The rhodium(III) chloride hydrate (RhCl_3_·xH_2_O, trace metals basis, 99.95%), sodium borohydride NaBH_4_, (ReagentPlus, 99%), iron(III) chloride hexahydrate (FeCl_3_·6H_2_O, American Chemical Society (ACS) reagent, 97%), hydrochloric acid (HCI, ACS reagent, 37%), sodium hydroxide (NaOH, ACS reagent, ≥97.0%), urea (CH_4_N_2_O, ACS reagent, 99.0%–100.5%), and Whatman qualitative filter paper (Grade 5, diameter: 110 mm) were all purchased from Sigma-Aldrich (Sigma-Aldrich Chemical Co., St. Louis, MO, USA).

### 2.2. Characterization

The content of the elements in the catalyst was measured using an Optima 4300DV ICP-OES (PerkinElmer Inc., Waltham, MA, USA). SEM images were acquired by a Tescan MAIA3 XMU microscope (Tescan, Brno, Kohoutovice, Czech Republic). The surface morphology and particle size analysis of the catalyst were characterized by TEM (Jeol-2100F; Jeol Ltd., Akishima, Tokyo, Japan) at an accelerating voltage of 200 kV. P-XRD patterns were recorded using a SmartLab X-ray diffractometer (Rigaku Corp., Tokyo, Japan) with Cu K_α_ radiation (λ = 1.5418 Å) operating at 40 kV and 55 mA. The surface composition and chemical states of the catalyst components were measured using an ESCA-LAB 250xi XPS (Thermo Fisher Scientific Inc., Waltham, MA, USA) equipped with an Al K_α_ X-ray excitation source. FTIR was performed using a Shimadzu IRTracer-100 spectrometer (Shimadzu Corp., Kyoto, Japan).

### 2.3. Synthesis of the g-C_3_N_4_

The g-C_3_N_4_ synthesis was carried out according to the method in the literature [24]. The starting material, urea (CH_4_N_2_O), was put into a high-quality alumina crucible and placed in a muffle furnace. Then, the muffle furnace was heated at a rate of 2 °C/min, and the sample was kept at 550 °C for 3 h. After cooling to room temperature, the obtained yellow powder was stored in a desiccator, to be used in the catalytic studies.

### 2.4. Preparation of the RhFe/g-C_3_N_4_ NPs

First, 150 mg of g-C_3_N_4_ was added to 10 mL of H_2_O and ultrasonicated for 15 min to form a well-dispersed suspension. Then, RhCl_3_·xH_2_O (0.015 mmol) and FeCl_3_·6H_2_O (0.014 mmol) aqueous solution were added, and the mixture was stirred with a magnetic stirrer at 700 rpm for 3 h. Next, NaBH_4_ (0.49 mmol) was added dropwise to the mixed solution at the molar ratio metal(s): NaBH_4_ = 1:15. The final mixture was washed with water/ethanol and filtered with Whatman No. 5 filter paper. The obtained sample was dried in an oven at 80 °C for 1 h. The dried sample was then stored in a desiccator for use in the catalytic experiment.

### 2.5. Catalytic hydrolysis of NaBH_4_ catalyzed by RhFe/g-C_3_N_4_

First, 50.0 mg of the RhFe/g-C_3_N_4_ catalyst was added to 4.0 mL of deionized water in a 30-mL jacketed Schlenk flask and placed in a thermostat water bath. The mixture was then stirred at 700 rpm and maintained at the desired temperature for 15 min to reach thermal equilibrium. Subsequently, the jacketed Schlenk flask was sealed with an airtight septum to initiate the reaction, followed by injecting 1.0 mL of NaBH_4_ solution (1 mmol) into the system using an airtight syringe. The volume of H_2_ released during the catalytic hydrolysis of NaBH_4_ catalyzed by the RhFe/g-C_3_N_4_ catalyst was monitored by measuring the water-displacement method in a 200-cm^3^ gas burette [25–27]. Upon completion of the NaBH_4_ hydrolysis, the recorded gas volume (in mL) over time was imported into Origin 10.1 software (OriginLab Corp., Northampton, MA, USA) to generate corresponding graphs.

## 3. Results

The g-C_3_N_4_ was synthesized by keeping the starting material (urea, CH_4_N_2_O) in a muffle furnace at 550 °C for 3 h. Then, RhFe NPs were decorated on the g-C_3_N_4_ surface using the impregnation-reduction method following a previously reported procedure. The RhFe/g-C_3_N_4_ bimetallic catalyst was obtained as dark gray powder and determined using ICP-OES, SEM, TEM, P-XRD, XPS, and FTIR analyses.

**Figure 1:**
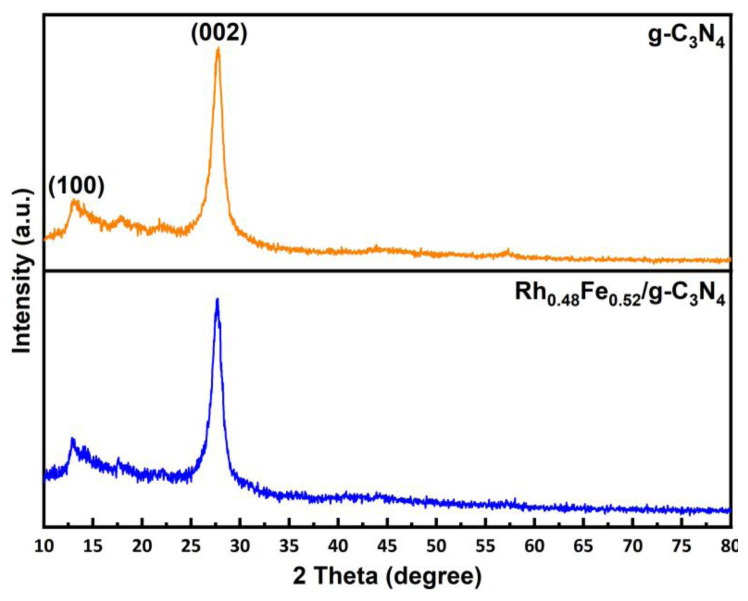
P-XRD spectra of the g-C_3_N_4_ and Rh_0.48_Fe_0.52_/g-C_3_N_4_ catalyst in the range of 2θ° of 10–80°.

ICP-OES analysis was used to determine the elemental composition of the RhFe/g-C_3_N_4_ catalyst. The molar composition of the Rh and Fe in the prepared RhFe/g-C_3_N_4_ catalyst was Rh_0.48_Fe_0.52_/g-C_3_N_4_ (0.38% wt Rh and 0.54% wt Fe). P-XRD analysis of the crystal and phase structures of the g-C_3_N_4_ and g-C_3_N_4_-supported Rh_0.48_Fe_0.52_ NPs are given in Figure 1. The Bragg diffraction peaks located at 12.96° (weak, corresponding to the in-plane structural motif of tris-triazine units) and 27.60° (strong, belonging to the interlayer stacking of the C_3_N_4_ layered structure) were attributed to the (100) and (002) crystal planes of the g-C_3_N_4_, respectively. These two Bragg diffraction peaks were consistent with g-C_3_N_4_, as reported in the literature [28,29]. The P-XRD pattern in Figure 1 shows that the crystal structure of the g-C_3_N_4_ in the Rh_0.48_Fe_0.52_/g-C_3_N_4_ catalyst was not affected by the decoration of the Rh_0.48_Fe_0.52_ NPs. Moreover, the Bragg diffraction peaks in the P-XRD corresponding to the Rh_0.48_Fe_0.52_ NPs could not be observed due to the low content of the Rh_0.48_Fe_0.52_ NPs (<5 wt%) and the good dispersion [30].

**Figure 2:**
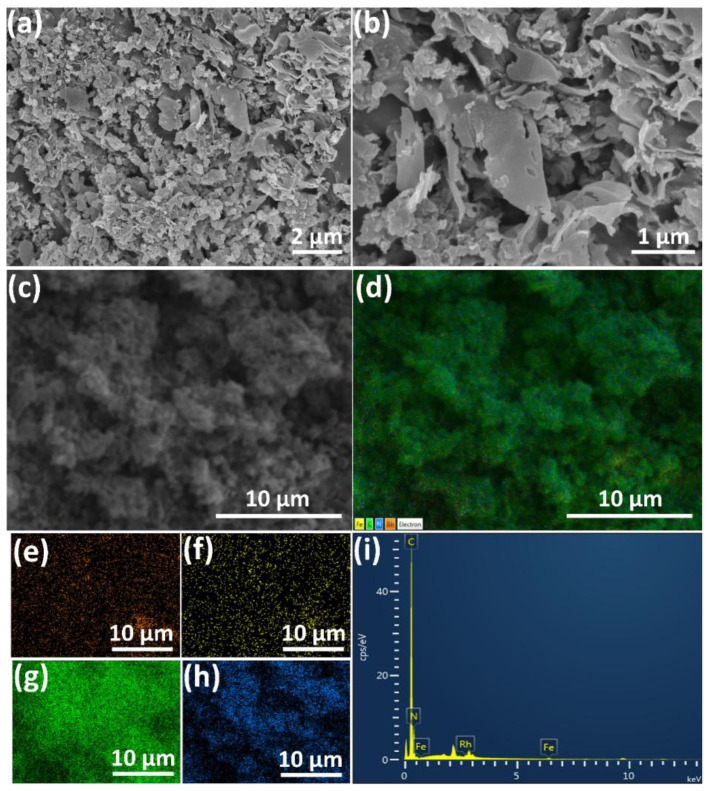
(a–c) SEM images, (d) combined (Rh, Fe, C, and N), (e) Rh, (f) Fe, (g) C, (h) N EDX elemental mapping, and (i) EDX spectrum of the Rh_0.48_Fe_0.52_/g-C_3_N_4_.

The SEM images (Figures 2a–2c) and their elemental composition mapping were used to analyze the provided information regarding the surface topography of the catalyst. The elemental mapping given in Figures 2d–2h shows that the Rh, Fe, C, and N in the Rh_0.48_Fe_0.52_/g-C_3_N_4_ catalyst were distributed homogeneously without any accumulation. Moreover, the SEM-EDX spectrum taken from a certain area confirmed the existence of the four elements (Figure 2i).

**Figure 3:**
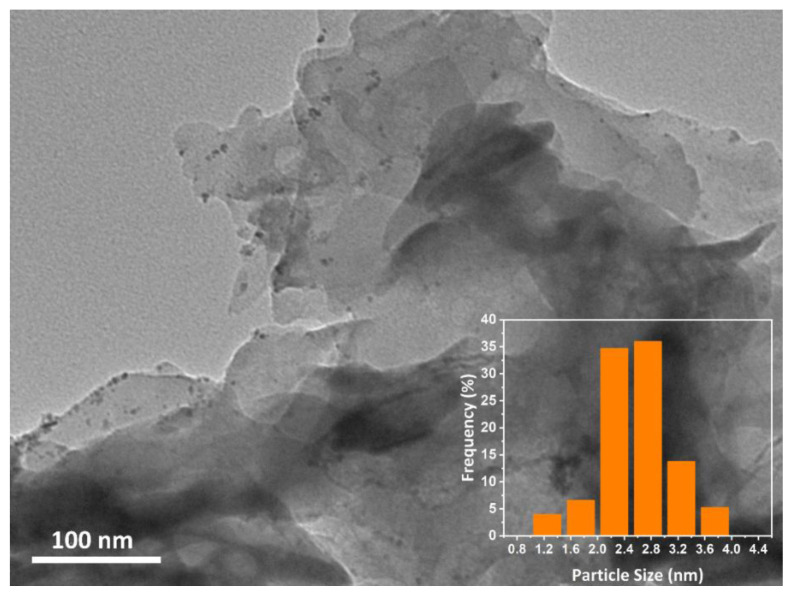
TEM image and average particle size histogram of the Rh_0.48_Fe_0.52_/g-C_3_N_4_.

At the same time, TEM analysis was used to determine the distribution and size of the NPs. Based on the TEM image (Figure 3) analysis conducted using ImageJ (US National Institutes of Health, Bethesda, MD, USA), the sample revealed approximately >94 RhFe NPs decorated on the g-C_3_N_4_ surface. The analysis further yielded an average particle size of 2.68 ± 0.63 nm (inset, Figure 3), indicating a uniform distribution of RhFe NPs with a relatively small size.

**Figure 4:**
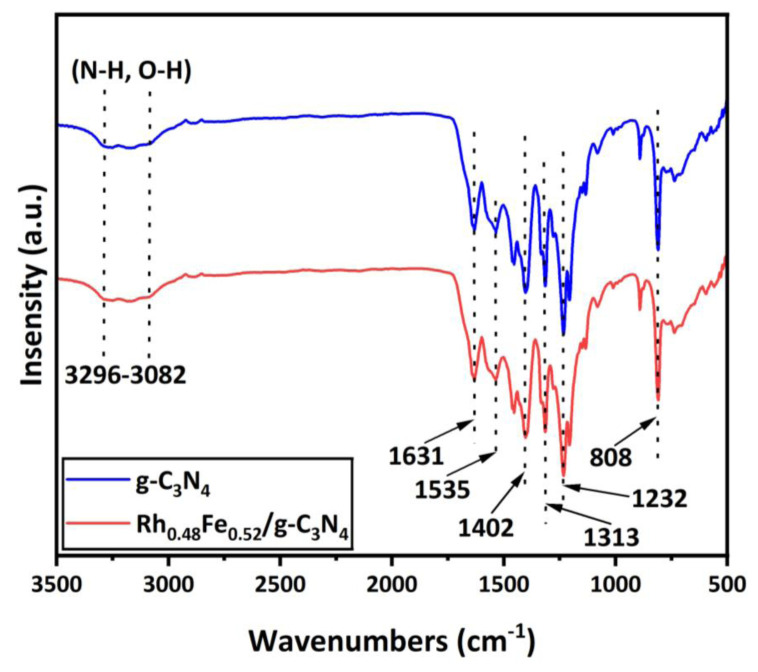
FTIR spectra of the g-C_3_N_4_ and Rh_0.48_Fe_0.52_/g-C_3_N_4_ catalyst.

FTIR analysis was used to investigate the chemical bonding structures of the g-C_3_N_4_ and Rh_0.48_Fe_0.52_/g-C_3_N_4_ samples (Figure 4). The characteristic peaks detected at approximately 1232, 1313, 1402, 1535, and 1631 cm^−1^ corresponded to the vibrations of C–N [31,32]. The peak at 808 cm^−1^ belonged to the individual breathing mode of triazine rings [33]. Moreover, the broad bands at 3082–3296 cm^−1^ were attributed to N–H and O–H stretching vibration modes [34,35]. Furthermore, the structure of the g-C_3_N_4_ was not affected due to the decoration of the Rh_0.48_Fe_0.52_ NPs.

**Figure 5:**
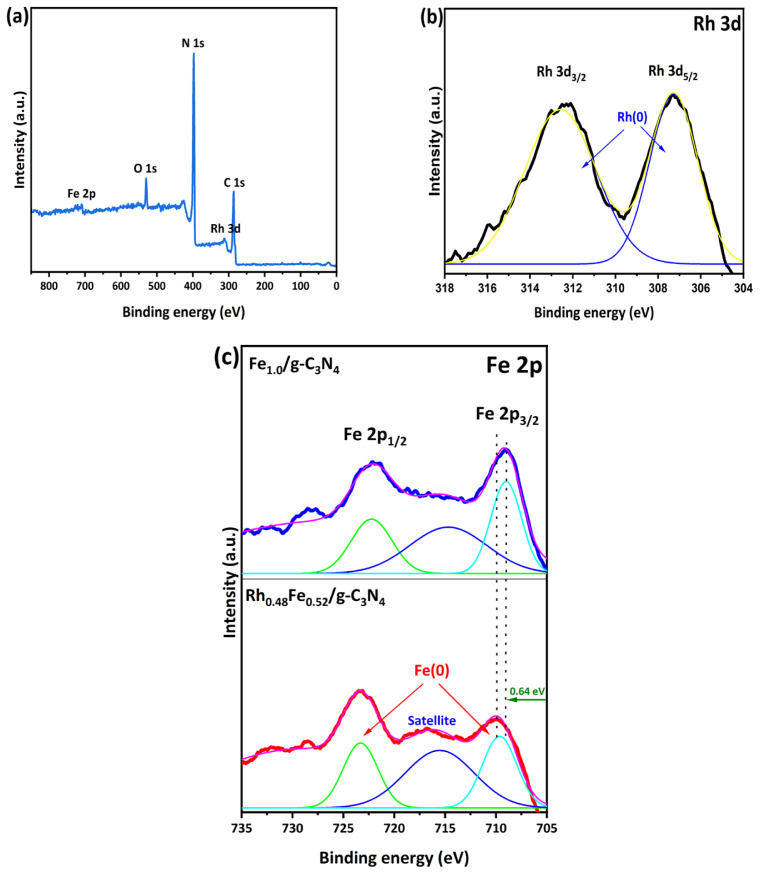
(a) XPS surface and HR XPS spectra of (b) Rh 3d and (c) Fe 2p for the Rh_0.48_Fe_0.52_/g-C_3_N_4_ catalyst.

**Figure 6:**
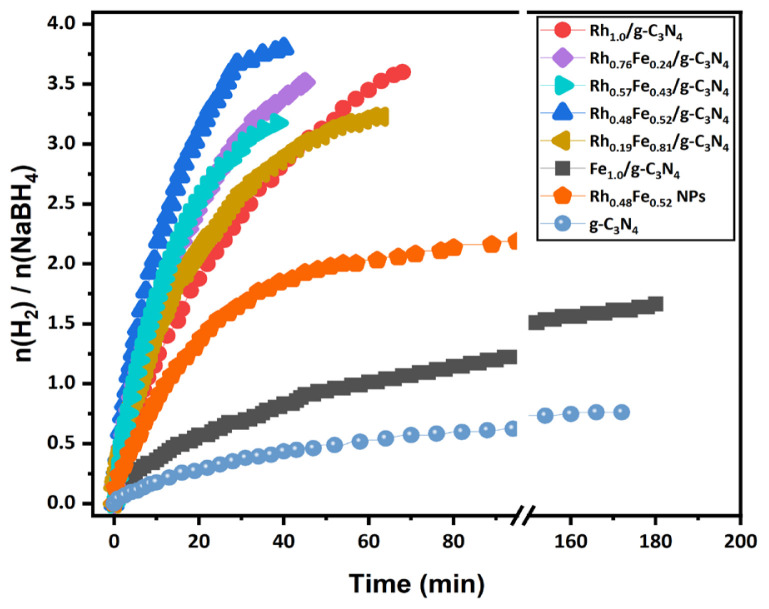
H_2_ produced kinetics curves of the different mono and bimetallic catalysts for the hydrolysis of NaBH_4_ at 298 K.

XPS analysis was conducted to elucidate the composition and chemical state of the Rh_0.48_Fe_0.52_/g-C_3_N_4_ catalyst. As depicted in Figure 5a, the surface XPS spectrum exhibited signals attributed to Rh, Fe, C, N, and O, affirming the successful attachment of Rh and Fe to the g-C_3_N_4_ surface. In the high-resolution (HR) Rh 3d XPS spectrum given in Figure 5b, the two central peaks at 307.27 and 312.58 eV are the Rh 3d_5/2_ and Rh 3d_3/2_ peaks of Rh(0), respectively [36]. The HR Fe 2p core-level spectrum of the Fe 2p_3/2_ exhibits two peaks at 709.0 and 722.2 eV associated with the Fe 2p_3/2_ and Fe 2p_1/2_ peak of Fe(0) for Rh_0.48_Fe_0.52_/g-C_3_N_4_ (Figure 5c) [37,38]. The binding energies of Fe(0) 2p_3/2_ and Fe(0) 2p_1/2_ for the Rh_0.48_Fe_0.52_/g-C_3_N_4_ exhibit an upshift of ~0.64 eV compared to Fe_1.0_/g-C_3_N_4_. This indicates that there was electron transfer between the Rh and Fe. This can be corroborated by examining Figure 6. The catalysts consisting of only Rh_1.0_/g-C_3_N_4_ and Fe_1.0_/g-C_3_N_4_ had much lower catalytic activities toward NaBH_4_ hydrolysis. However, the Rh_0.48_Fe_0.52_/g-C_3_N_4_ bimetallic catalyst had much better catalytic activity.

The molar metal composition (Rh/Fe) impacted the performance of RhFe/g-C_3_N_4_ in the hydrolysis of NaBH_4_. H_2_ production via the hydrolysis of NaBH_4_ was evaluated using mono/bimetallic catalysts (Rh_1.0_/g-C_3_N_4_, Fe_1.0_/g-C_3_N_4_, Rh_x_Fe_1-x_/g-C_3_N_4_) prepared in NaBH_4_ aqueous solution at 298 K. Figure 6 shows the time-dependent H_2_ evaluation graph in NaBH_4_ hydrolysis catalyzed by RhFe/g-C_3_N_4_ catalysts with different Rh/Fe molar ratios. The graph shows that the Rh_1.0_/g-C_3_N_4_ and Fe_1.0_/g-C_3_N_4_ catalysts produce 3.6 moles of H_2_ in 68 min and 1.66 moles of H_2_ in 180 min in the NaBH_4_ hydrolysis, which indicates that the Rh/g-C_3_N_4_ and Fe/g-C_3_N_4_ catalysts alone could not release 4 moles of H_2_ from NaBH_4_ hydrolysis. As the molar ratio between the Rh and Fe in the Rh_x_Fe_1-x_/g-C_3_N_4_ catalyst changed, the catalytic efficiency in the NaBH_4_ hydrolysis increased. The catalytic activities obtained in the bimetallic Rh_0.48_Fe_0.52_/g-C_3_N_4_ catalysts were compared to the monometallic Rh_1.0_/g-C_3_N_4_ and Fe_1.0_/g-C_3_N_4_ catalysts in terms of the initial turnover frequency (TOF) values. Among the bimetallic catalysts in the graph, the optimized Rh_0.48_Fe_0.52_/g-C_3_N_4_ catalyst provided the highest activity in the hydrolysis of NaBH_4_, with an initial TOF value of $33.04\;{\text{mol}}_{{\text{H}}_{2}}\;{\text{mol}}_{\text{metal}}^{-1}\;{\text{min}}^{-1}$ ~3.9 moles of H_2_ in 40 min. Moreover, the Rh_0.48_Fe_0.52_/g-C_3_N_4_ catalyst exhibited a TOF value of $33.04\;{\text{mol}}_{{\text{H}}_{2}}\;{\text{mol}}_{\text{metal}}^{-1}\;{\text{min}}^{-1}$, which was higher than those of the ${\text{Rh}}_{1.0}/\text{g-}{\text{C}}_{3}{\text{N}}_{4}\;(18.21\;{\text{mol}}_{{\text{H}}_{2}}\;{\text{mol}}_{\text{metal}}^{-1}\;{\text{min}}^{-1}),\;{\text{Fe}}_{1.0}/\text{g-}{\text{C}}_{3}{\text{N}}_{4}\;(4.93\;{\text{mol}}_{{\text{H}}_{2}}\;{\text{mol}}_{\text{metal}}^{-1}\;{\text{min}}^{-1})$ and colloidal Rh_0.48_Fe_0.52_ NPs without $\text{g-}{\text{C}}_{3}{\text{N}}_{4}\;(12.21\;{\text{mol}}_{{\text{H}}_{2}}\;{\text{mol}}_{\text{metal}}^{-1}\;{\text{min}}^{-1})$. The effect of the g-C_3_N_4_ on NaBH_4_ hydrolysis was also investigated without impregnation of the Rh_0.48_Fe_0.52_ NPs, and it was observed that g-C_3_N_4_ exhibited very low catalytic activity compared to the Rh_0.48_Fe_0.52_/g-C_3_N_4_ catalyst.

**Figure 7:**
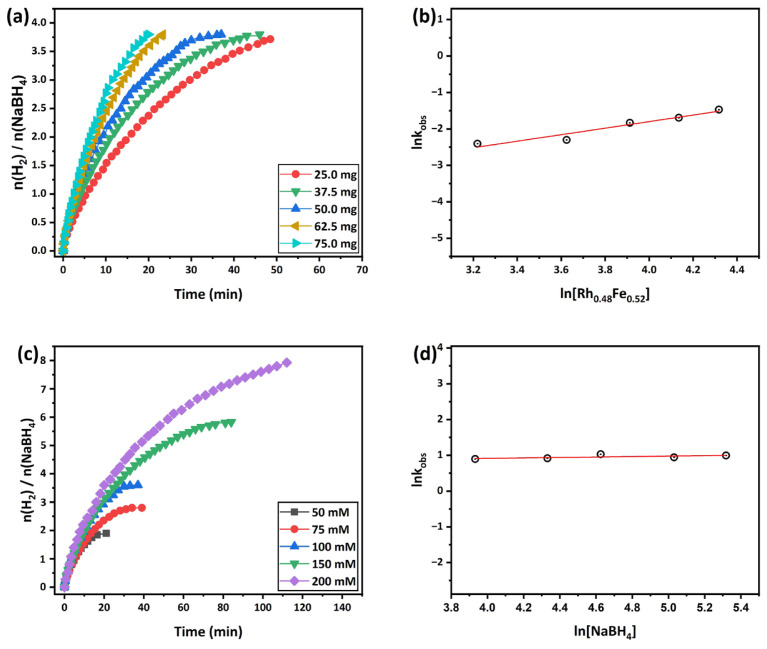
(a) H_2_ produced kinetics curves of different catalyst amount for the hydrolysis of NaBH_4_ catalyzed by the Rh_0.48_Fe_0.52_/g-C_3_N_4_ at 298 K, (b) logarithmic plot of the H_2_ produced rate versus the amount of catalyst, (c) H_2_ produced kinetics curves of the different NaBH_4_ concentrations for the hydrolysis of NaBH_4_ catalyzed by the Rh_0.48_Fe_0.52_/g-C_3_N_4_ at 298 K, and (d) logarithmic plot of the H_2_ produced rate versus the NaBH_4_ concentrations.

The effect of the amount of [Rh_0.48_Fe_0.52_] on the hydrolysis of NaBH_4_ over the Rh_0.5_Fe_0.5_/g-C_3_N_4_ catalyst was investigated across amounts of [Rh_0.48_Fe_0.52_] ranging from 25 to 75 mg ([NaBH_4_] = 100 mM, 298 K). Figure 7a depicts the volumetric H_2_ released over time during hydrolysis of the NaBH4 on the Rh_0.48_Fe_0.52_/g-C_3_N_4_ catalyst at varying amounts [Rh_0.48_Fe_0.52_]. As seen in the graph, the hydrolysis time of NaBH_4_ increased as the amount of [Rh_0.48_Fe_0.52_] increased from 25 to 75 mg (25, 37.5, 50, 62.5, and 75.0 mg amount of catalyst correspond to 0.95, 1.42, 1.9, 2.37, and 2.84 mM RhFe concentrations). The H_2_ evolution rates for the different amounts of [Rh_0.48_Fe_0.52_] were determined by examining the linear parts of each plot in Figure 7b. The resulting linear part gave a slope of 0.94, indicating that the catalytic process of NaBH_4_ hydrolysis by Rh_0.48_Fe_0.52_/g-C_3_N_4_ adhered to first-order kinetics. Figure 7c shows the effect of different [NaBH_4_] concentrations on the hydrolysis of NaBH_4_ catalyzed by Rh_0.48_Fe_0.52_/g-C_3_N_4_ (catalyst = 50 mg, 298 K]). The slope was calculated as 0.06 from the logarithmic graph of H_2_ evolution rates obtained from each graph in Figure 7d. This indicates that the hydrolysis of NaBH_4_ catalyzed by Rh_0.48_Fe_0.52_/g-C_3_N_4_ was a zero-order reaction related to the [NaBH_4_] concentration. The temperature effect on the H_2_ evolution rate in the NaBH_4_ hydrolysis catalyzed by Rh_0.48_Fe_0.52_/g-C_3_N_4_ was also examined (catalyst = 50 mg, [NaBH_4_] = 100 mM).

**Figure 8:**
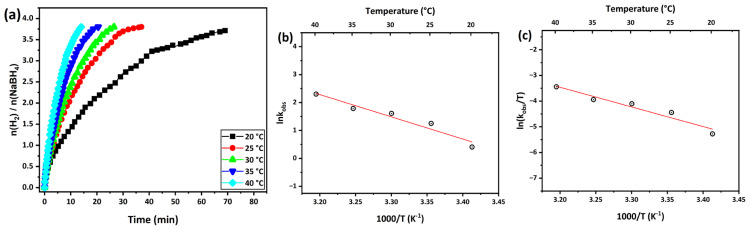
(a) H_2_ produced kinetics curves of the different temperatures for the hydrolysis of NaBH_4_ catalyzed by the Rh_0.48_Fe_0.52_/g-C_3_N_4_, (b) Arrhenius plot, and (c) Eyring-Polanyi plot.

Figure 8 shows the volumetric H_2_ release versus time plot for hydrolysis of the NaBH_4_ on the Rh_0.48_Fe_0.52_/g-C_3_N_4_ catalyst at different temperatures (20, 25, 30, 35, and 40 °C). In the graph, the H_2_ evolution rate also increased with temperature, which shows that temperature seriously impacted the H_2_ formation rate in hydrolysis of the NaBH_4_. The activation energy (*E*_a_^#^) is a critical metric for investigating the catalytic process of NaBH_4_ hydrolysis. The *E*_a_^#^ value for the hydrolysis reaction catalyzed by the optimized Rh_0.48_Fe_0.52_/g-C_3_N_4_ bimetallic catalyst was determined using the Arrhenius equation. In Figures 8b and 8c, the rate constants calculated from the linear part of each graph were transferred to the Arrhenius and Eyring equations and calculated as *E*_a_^#^ 66.20 kJ mol^−1^, activation enthalpy (Δ*H*^#^) 63.68 kJ mol^−1^, and activation entropy (Δ*S*^#^) −149.53 J mol^−1^ K^−1^.

The *E*a^#^ for the Rh_0.5_Fe_0.5_/g-C_3_N_4_ was lower than most of the catalysts reported in Table, indicating that this catalyst can remarkably reduce the energy barrier for NaBH_4_ hydrolysis, thus facilitating the formation of H_2_ from NaBH_4_ hydrolysis under mild conditions.

**Figure 9:**
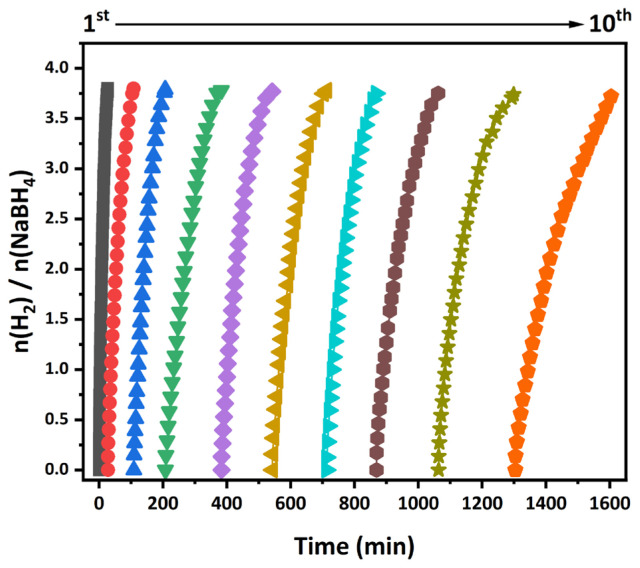
Recyclability performance of the NaBH_4_ catalyzed by the Rh_0.48_Fe_0.52_/g-C_3_N_4_ catalyst up to the 10th cycle at 298 K.

Moreover, the stability of the catalyst is significant for use in large-scale industrial production. The recyclability performance of the Rh_0.48_Fe_0.52_/g-C_3_N_4_ catalyst for the hydrolysis of NaBH_4_ up to the 10th catalytic cycle is given in Figure 9. Following completion of the 1st cycle, fresh NaBH_4_ solution was added to the reaction medium, and catalytic testing continued through the 10th cycle. The Rh_0.48_Fe_0.52_/g-C_3_N_4_ catalyst catalyzed the hydrolysis of NaBH_4_ to produce about ~4 equivalent of H_2_ per mole of NaBH_4_. As the number of cycles increased, the conversion time of the NaBH_4_ also increased slightly. This indicates a decrease in activity, which caused the accumulation of NaBO_2_ [51], a byproduct formed in the solution with each addition of NaBH_4_ to the system. Therefore, this shows that it blocked the catalyst’s active sites and prevented contact between the catalyst and the NaBH_4_ solution.

**Figure 10:**
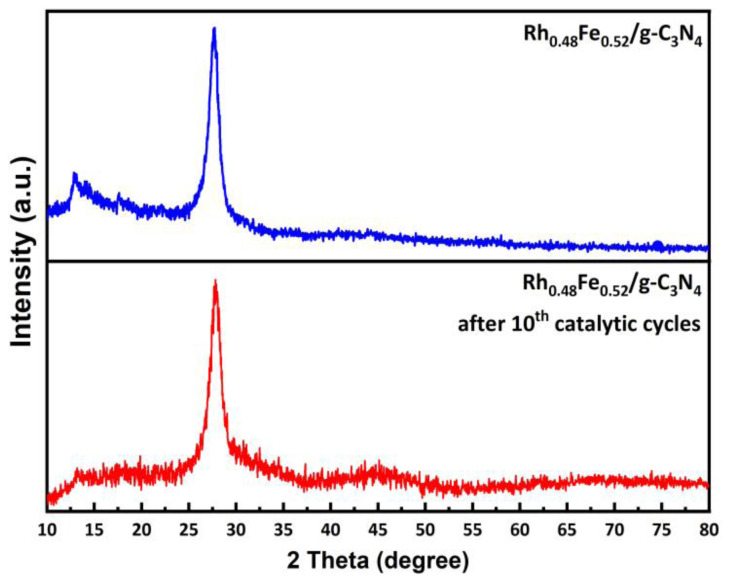
P-XRD spectra of after 10th catalytic cycle for the Rh_0.48_Fe_0.52_/g-C_3_N_4_ and Rh_0.48_Fe_0.52_/g-C_3_N_4_ catalyst in the range of 2θ° of 10–80°.

The P-XRD pattern of the Rh_0.48_Fe_0.52_/g-C_3_N_4_ catalyst taken after 10 cycles (Figure 10) was the same as that of the Rh_0.48_Fe_0.52_/g-C_3_N_4_, indicating that the crystallinity of the solid support material (g-C_3_N_4_) was maintained after 10 cycles.

## 4. Discussion

The preparation of a RhFe/g-C_3_N_4_ catalyst via a simple and reproducible impregnation-reduction method in a 4-h procedure was presented. The optimized Rh_0.48_Fe_0.52_/g-C_3_N_4_ catalyst exhibited outstanding performance with an initial TOF of 33.04 mol_H2_ mol^−1^_metal_h^−1^ and an H_2_ generation rate (HGR) of $4214.02\;\text{mL }{\text{min}}^{-1}\;{\text{g}}_{\text{cat}}^{-1}$ at 298 K. The catalytic activity of the Rh_0.48_Fe_0.52_/g-C_3_N_4_ in the hydrolysis of NaBH_4_ was evaluated under different temperatures, concentrations of [RhFe NPs] and [NaBH_4_], and recyclability conditions. The activation parameters (*E*_a_^#^, Δ*H*^#^, Δ*S*^#^) for hydrolysis of the NaBH_4_ catalyzed by Rh_0.48_Fe_0.52_/g-C_3_N_4_ were calculated as 66.20 kJ mol^−1^, 63.68 kJ mol^−1^, and −149.53 kJ mol^−1^ K^−1^, respectively. Moreover, the resulting Rh_0.48_Fe_0.52_/g-C_3_N_4_ catalyst was recyclable and retained 80.72% of the initial activity even after 10 runs. These findings highlight the potential of Rh_0.48_Fe_0.52_/g-C_3_N_4_ catalysts for efficient H_2_ production, paving the way for their application in fuel cell-based H_2_ economies.

## Figures and Tables

**Table:** TOF and *E*a^#^ values of the catalysts tested in the hydrolysis of NaBH_4_.

	Catalyst	$\text{HGR }(\text{mL }{\text{min}}^{-1}\;{\text{g}}_{\text{cat}}^{-1})$	Ea^#^ (kJ mol^−1^)	Reference
**1**	Pd/Co_3_O_4_	2109	65.82	[39]
**2**	Fe/SiO_2_	130	59	[40]
**3**	Fe_2_O_3_@BS	286	39.59	[41]
**4**	Ru-RuO_2_/C	2800	-	[42]
**5**	Ru_4.7_/Co-Sm_2_O_3_	9636	53.2	[43]
**6**	RuCo/C	3420	29.3	[44]
**7**	Cu-Co-B	2120	49.6	[45]
**8**	Fe-B	59	64.26	[46]
**9**	Ru-9.0/ZIF-67	5520	39	[47]
**10**	Co@NHC	1515.4	14.8	[48]
**11**	FeCo_2_O_4_/CC	2251	45.0	[49]
**12**	FeCuCoO_x_	1380	16.3	[50]
**13**	**Rh** **_0.48_** **Fe** **_0.52_** **/g-C** **_3_** **N** **_4_**	**4214.02**	**66.20**	**This study**

## References

[1] Long B Chen J Sharshir SW Ibrahim L Zhou W The mechanism and challenges of cobalt-boron-based catalysts in the hydrolysis of sodium borohydride Journal of Material Chemistry A 2024 12 10 5606 5625 10.1039/D3TA07308D

[2] Pope F Jonk J Fowler M Lan PCM Geels NJ From shrimp balls to hydrogen bubbles: borohydride hydrolysis catalysed by flexible cobalt chitosan spheres Green Chemistry 2023 25 14 5727 5734 10.1039/D3GC00821E

[3] Netskina OV Bulakov VE Sukhorukov DA Ozerova AM Prosvirin IP Ferromagnetic “nickel core–cobalt shell” catalysts for NaBH_4_ hydrolysis New Journal of Chemistry 2024 48 7 3304 3315 10.1039/D3NJ04579J

[4] Çetin MB Top T Yurderi M Zahmakıran M Rakap M Tungsten(VI) oxide-supported nickel/silver nanoparticles for photocatalytic hydrogen evolution from ammonia-borane International Journal of Hydrogen Energy 2024 72 60 73 10.1016/j.ijhydene.2024.05.361

[5] Liu X Sun W Chen J Wen Z Controllable electrochemical liberation of hydrogen from sodium borohydride Angewandte Chemie International Edition 2024 136 4 e202317313 10.1002/anie.202317313 38055203

[6] Ekinci A Genli N Şahin Ö Baytar O Facile “Green” synthesis of a novel Co–W–B catalyst from Rheum ribes shell extract and its effect on sodium borohydride hydrolysis: Kinetic mechanism International Journal of Hydrogen Energy 2024 51 796 808 10.1016/j.ijhydene.2023.07.069

[7] Sun Q Wang N Xu Q Yu J Nanopore-supported metal nanocatalysts for efficient hydrogen generation from liquid-phase chemical hydrogen storage materials Advanced Materials 2020 32 44 2001818 10.1002/adma.202001818 32638425

[8] Demirci UB Miele P Sodium borohydride versus ammonia borane, in hydrogen storage and direct fuel cell applications Energy & Environmental Science 2009 2 6 627 637 10.1039/B900595A

[9] Zhang H Zhang L Rodríguez-Pérez IA Miao W Chen K Carbon nanospheres supported bimetallic Pt-Co as an efficient catalyst for NaBH_4_ hydrolysis Applied Surface Science 2021 540 148296 10.1016/j.apsusc.2020.148296

[10] Shang N Zhou X Feng C Gao S Wu Q Synergetic catalysis of NiPd nanoparticles supported on biomass-derived carbon spheres for hydrogen production from ammonia borane at room temperature International Journal of Hydrogen Energy 2017 42 9 5733 5740 10.1016/j.ijhydene.2016.11.192

[11] Al-Thabaiti SA Khan Z Malik MA Bimetallic Ag-Ni nanoparticles as an effective catalyst for hydrogen generation from hydrolysis of sodium borohydride International Journal of Hydrogen Energy 2019 44 31 16452 16466 10.1016/j.ijhydene.2019.04.240

[12] Mori K Miyawaki K Yamashita H Ru and Ru-Ni nanoparticles on TiO_2_ support as extremely active catalysts for hydrogen production from ammonia-borane ACS Catalysis 2016 6 5 3128 3135 10.1021/acscatal.6b00715

[13] Li YT Zhang XL Peng ZK Liu P Zheng XC Hierarchical porous g-C_3_N_4_ coupled ultrafine RuNi alloys as extremely active catalysts for the hydrolytic dehydrogenation of ammonia borane ACS Sustainable Chemistry & Engineering 2020 8 22 8458 8468 10.1021/acssuschemeng.0c03009

[14] Pradhan MR Nanda BB Subhadarshini A Panda L Nanda B Enhanced catalytic reductive hydrogenation of an organic dye by Ag decorated graphitic carbon nitride modified MCM-41 RSC Advances 2024 14 2 1072 1081 10.1039/D3RA05608B 38174238 PMC10759964

[15] Singh Chouhan R Jerman I Heath D Bohm S Gandhi S Emerging tri-s-triazine-based graphitic carbon nitride: a potential signal-transducing nanostructured material for sensor applications Nano Select 2021 2 4 712 743 10.1002/nano.202000228

[16] Niu P Dai J Zhi X Xia Z Wang S Photocatalytic overall water splitting by graphitic carbon nitride InfoMat 2021 3 9 931 961 10.1002/inf2.12219

[17] Idris AO Oseghe EO Msagati TAM Kuvarega AT Feleni U Graphitic carbon nitride: a highly electroactive nanomaterial for environmental and clinical sensing Sensors 2020 20 20 5743 10.3390/s20205743 33050361 PMC7600177

[18] Zhang Y Liu D Shi J Chen P Zong S (Oxy)nitride heterojunction-strengthened separation of photogenerated carriers in g-C_3_N_4_ towards enhanced photocatalytic H_2_ evolution Applied Catalysis A: General 2022 643 118746 10.1016/j.apcata.2022.118746

[19] Li Y Chen C Chen X Zang J MoO_3_/g-C_3_N_4_ heterostructure for degradation of organic pollutants under visible light irradiation: high efficiency, general degradation and Z-scheme degradation mechanism Ceramics International 2021 47 23 33697 33708 10.1016/j.ceramint.2021.08.279

[20] Li Y Zhu S Kong X Liang Y Li Z In situ synthesis of a novel Mn_3_O_4_/g-C_3_N_4_ p-n heterostructure photocatalyst for water splitting Journal of Colloid and Interface Science 2021 586 778 784 10.1016/j.jcis.2020.11.002 33198987

[21] Hu J Zhang P Yang T Cai Y Qu J Screen superior ultra-thin g-C_3_N_4_ material for photocatalytic in-situ H_2_O_2_ production to remove tetracycline Applied Surface Science 2022 576 151841 10.1016/j.apsusc.2021.151841

[22] Liu S Liu JX Yang M Zhang XL Zheng XC Ultrafine Pd nanoparticles stabilized on magnetic Fe_3_O_4_@SiO_2_-g-C_3_N_4_ composites for the hydrolytic dehydrogenation of ammonia borane International Journal of Hydrogen Energy 2020 45 55 30511 30520 10.1016/j.ijhydene.2020.08.064

[23] Lu R Hu M Xu C Wang Y Zhang Y Hydrogen evolution from hydrolysis of ammonia borane catalyzed by Rh/g-C_3_N_4_ under mild conditions International Journal of Hydrogen Energy 2018 43 14 7038 7045 10.1016/j.ijhydene.2018.02.148

[24] Chava RK Kang M Ordered and carbon-doped porous polymeric graphitic carbon nitride nanosheets toward enhanced visible light absorption and efficient photocatalytic H_2_ evolution Nanoscale 2023 15 45 18347 18358 10.1039/D3NR04270G 37921504

[25] Xu F Huang W Wang Y Astruc D Liu X Efficient and controlled H_2_ release from sodium formate Inorganic Chemistry Frontiers 2022 9 14 3514 3521 10.1039/D2QI00774F

[26] Li G Wei N Wang Y Active clusters ensemble effect of bimetallic RuCo alloys for efficient hydrogen production from ammonia borane Applied Surface Science 2023 610 155459 10.1016/j.apsusc.2022.155459

[27] Yılmaz C Yıldırım HA Top T Yurderi M Zahmakıran M ZIF-8 decorated FeMo nanoparticles: H_2_ production from the catalytic hydrolysis of ammonia-borane Environmental and Progress & Sustainable Energy 2024 43 5 e14439 10.1002/ep.14439

[28] Ding X Gao R Chen Y Wang H Liu Y Carbon vacancies in graphitic carbon nitride-driven high catalytic performance of Pd/CN for phenol-selective hydrogenation to cyclohexanone ACS Catalysis 2024 14 5 3308 3319 10.1021/acscatal.3c05625

[29] Ismael M Construction of novel Ru-embedded bulk g-C_3_N_4_ photocatalysts toward efficient and sustainable photocatalytic hydrogen production Diamond & Related Materials 2024 144 111024 10.1016/j.diamond.2024.111024

[30] Han C Lu Y Zhang J Ge L Li Y Novel PtCo alloy nanoparticle decorated 2D g-C_3_N_4_ nanosheets with enhanced photocatalytic activity for H_2_ evolution under visible light irradiation Journal of Materials Chemistry A 2015 3 46 23274 23282 10.1039/C5TA05370F

[31] Kim M Hwang S Yu JS Novel ordered nanoporous graphitic C_3_N_4_ as a support for Pt–Ru anode catalyst in direct methanol fuel cell Journal of Materials Chemistry 2007 17 17 1656 1659 10.1039/B702213A

[32] Mo Z She X Li Y Liu L Huang L Synthesis of g-C_3_N_4_ at different temperatures for superior visible/UV photocatalytic performance and photoelectrochemical sensing of MB solution RSC Advances 2015 5 123 101552 101562 10.1039/C5RA19586A

[33] Thanh Truc NT Hanh NT Nguyen MV Le Chi NTP Noi NV Novel direct Z-scheme Cu_2_V_2_O_7_/g-C_3_N_4_ for visible light photocatalytic conversion of CO_2_ into valuable fuels Applied Surface Science 2018 457 968 974 10.1016/j.apsusc.2018.07.034

[34] Qi K Zada A Yang Y Chen Q Khataee A Design of 2D–2D NiO/g-C_3_N_4_ heterojunction photocatalysts for degradation of an emerging pollutant Research on Chemical Intermediates 2020 46 12 5281 5295 10.1007/s11164-020-04262-0

[35] Narkbuakaew T Sujaridworakun P Synthesis of tri-s-triazine based g-C_3_N_4_ photocatalyst for cationic rhodamine B degradation under visible light Topics in Catalysis 2020 63 11–14 1086 1096 10.1007/s11244-020-01375-z

[36] Zhu JY Li FM Yao L Han CC Li SN In situ bubble template-assisted synthesis of phosphonate-functionalized Rh nanodendrites and their catalytic application CrystEngComm 2017 19 21 2946 2952 10.1039/C7CE00606C

[37] Paksoy A Kurtoğlu-Öztulum SF Yağcı MB Balcı-Çağıran Ö Low-cost and reusable iron- and nickel-based metal boride nanoparticles for efficient catalytic hydrolysis of sodium borohydride International Journal of Hydrogen Energy 2022 47 87 36898 36913 10.1016/j.ijhydene.2022.08.269

[38] Guan K Wang L Huang L Lei W Jia Q Synthesis and high catalytic activity of ISOBAM-104 stabilized Fe colloidal catalysts for hydrogen generation Catalysis Today 2021 374 20 28 10.1016/j.cattod.2020.10.028

[39] Bozkurt G Özer A Yurtcan AB Development of effective catalysts for hydrogen generation from sodium borohydride: Ru, Pt, Pd nanoparticles supported on Co_3_O_4_ Energy 2019 180 702 713 10.1016/j.energy.2019.04.196

[40] Shih YJ Su CC Huang YH Lu MC SiO_2_-supported ferromagnetic catalysts for hydrogen generation from alkaline NaBH_4_ (sodium borohydride) solution Energy 2013 54 263 270 10.1016/j.energy.2013.01.063

[41] Duman S Kaya B Caf F Enez B Fincan SA Innovative hydrogen release from sodium borohydride hydrolysis using biocatalyst-like Fe_2_O_3_ nanoparticles impregnated on Bacillus simplex bacteria International Journal of Hydrogen Energy 2021 46 29 15410 15430 10.1016/j.ijhydene.2021.02.028

[42] Li Y Zhang Q Zhang N Zhu L Zheng J Ru–RuO_2_/C as an efficient catalyst for the sodium borohydride hydrolysis to hydrogen International Journal of Hydrogen Energy 2013 38 30 13360 13367 10.1016/j.ijhydene.2013.07.071

[43] Zhou S Yang Q Liu Y Cheng L Isimjan TT Electronic metal-support interactions for defect-induced Ru/Co-Sm2O3 mesosphere to achieve efficient NaBH_4_ hydrolysis activity Journal of Catalysis 2024 433 115491 10.1016/j.jcat.2024.115491

[44] Fiorenza R Scirè S Venezia AM Carbon supported bimetallic Ru-Co catalysts for H_2_ production through NaBH_4_ and NH_3_BH_3_ hydrolysis International Journal of Energy Research 2018 42 3 1183 1195 10.1002/er.3918

[45] Ding XL Yuan X Jia C Ma ZF Hydrogen generation from catalytic hydrolysis of sodium borohydride solution using Cobalt–Copper–Boride (Co–Cu–B) catalysts International Journal of Hydrogen Energy 2010 35 20 11077 11084 10.1016/j.ijhydene.2010.07.030

[46] Tuan TN Yi Y Lee JK Lee J Fe–B catalyst fabricated by hybrid capacitive adsorption–chemical reduction method and its application for hydrogen production from NaBH_4_ solution Catalysis Today 2013 216 240 245 10.1016/j.cattod.2013.05.024

[47] Tuan DD Lin KYA Ruthenium supported on ZIF-67 as an enhanced catalyst for hydrogen generation from hydrolysis of sodium borohydride Chemical Engineering Journal 2018 351 48 55 10.1016/j.cej.2018.06.082

[48] Tran DT Van HT Nguyen LH Quang NV Tsai YC Hierarchical porous cobalt nanoparticles encapsulated in heteroatom-doped hollow carbon as an enhancing multifunctional catalyst for hydrolysis of sodium borohydride and hydrogenation of bromate in water Surfaces and Interfaces 2024 48 104329 10.1016/j.surfin.2024.104329

[49] Hao S Yang L Cui L Lu W Yang Y Self-supported spinel FeCo_2_O_4_ nanowire array: an efficient non-noble-metal catalyst for the hydrolysis of NaBH_4_ toward on-demand hydrogen generation Nanotechnology 2016 27 46 46LT03 10.1088/0957-4484/27/46/46LT03 27734803

[50] Patil KN Prasad D Bhagyashree Manoorkar VK Nabgan W Engineered nano-foam of tri-metallic (FeCuCo) oxide catalyst for enhanced hydrogen generation via NaBH_4_ hydrolysis Chemosphere 2021 281 130988 10.1016/j.chemosphere.2021.130988 34289632

[51] Zhang H Xu G Zhang L Wang W Miao W Ultrafine cobalt nanoparticles supported on carbon nanospheres for hydrolysis of sodium borohydride Renewable Energy 2020 162 345 354 10.1016/j.renene.2020.08.031

